# Chromatic Pupillometry Methods for Assessing Photoreceptor Health in Retinal and Optic Nerve Diseases

**DOI:** 10.3389/fneur.2019.00076

**Published:** 2019-02-12

**Authors:** A. V. Rukmini, Dan Milea, Joshua J. Gooley

**Affiliations:** ^1^Programme in Neuroscience and Behavioural Disorders, Centre for Cognitive Neuroscience, Duke-NUS Medical School, Singapore, Singapore; ^2^Singapore National Eye Centre, Singapore Eye Research Institute, Singapore, Singapore; ^3^The Ophthalmology and Visual Sciences Academic Clinical Programme (EYE-ACP), SingHealth and Duke-NUS, Singapore, Singapore

**Keywords:** pupillometry, pupillary light reflex, melanopsin, retina, blind, optic nerve, glaucoma, blue light

## Abstract

The pupillary light reflex is mediated by melanopsin-containing intrinsically-photosensitive retinal ganglion cells (ipRGCs), which also receive input from rods and cones. Melanopsin-dependent pupillary light responses are short-wavelength sensitive, have a higher threshold of activation, and are much slower to activate and de-activate compared with rod/cone-mediated responses. Given that rod/cone photoreceptors and melanopsin differ in their response properties, light stimuli can be designed to stimulate preferentially each of the different photoreceptor types, providing a read-out of their function. This has given rise to chromatic pupillometry methods that aim to assess the health of outer retinal photoreceptors and ipRGCs by measuring pupillary responses to blue or red light stimuli. Here, we review different types of chromatic pupillometry protocols that have been tested in patients with retinal or optic nerve disease, including approaches that use short-duration light exposures or continuous exposure to light. Across different protocols, patients with outer retinal disease (e.g., retinitis pigmentosa or Leber congenital amaurosis) show reduced or absent pupillary responses to dim blue-light stimuli used to assess rod function, and reduced responses to moderately-bright red-light stimuli used to assess cone function. By comparison, patients with optic nerve disease (e.g., glaucoma or ischemic optic neuropathy, but not mitochondrial disease) show impaired pupillary responses during continuous exposure to bright blue-light stimuli, and a reduced post-illumination pupillary response after light offset, used to assess melanopsin function. These proof-of-concept studies demonstrate that chromatic pupillometry methods can be used to assess damage to rod/cone photoreceptors and ipRGCs. In future studies, it will be important to determine whether chromatic pupillometry methods can be used for screening and early detection of retinal and optic nerve diseases. Such methods may also prove useful for objectively evaluating the degree of recovery to ipRGC function in blind patients who undergo gene therapy or other treatments to restore vision.

## Introduction

The pupillary light reflex is routinely used to assess visual system function and optic nerve disease. As noted by the Greek physician Galen more than 1,800 years ago, poor vision is often characterized by a poor pupillary response to light (1). Until the end of the twentieth century, it was widely assumed that rod and cone photoreceptors that mediate image-forming vision were also responsible for the pupillary light reflex. In normally-sighted individuals, the threshold and spectral sensitivity of pupillary responses closely resembled visual responses (2–5), suggesting involvement of rod and cone photoreceptors. Additionally, pupillary light responses were abnormal in patients with loss of either rod or cone function (6, 7), and were altered in individuals with various forms of color-defective vision (8). Visual field defects were also generally well matched by pupillary field deficits using pupil perimetry (9). Together, these findings supported the conclusion that similar photoreceptor pathways were involved in the pupillary light reflex and image-forming vision. This view was turned on its head when it was discovered that the outer retina was not required for the pupil to respond to light.

Clyde Keeler's pioneering studies of “rodless mice” (gene symbol, *r*, or *rd*) in the 1920s foreshadowed work that led to the identification of photoreceptors in the inner retina. Keeler's *rd/rd* mice showed rapid loss of rods in early postnatal development, followed by secondary degeneration of cone photoreceptors (10). Despite showing behavioral and physiologic signs of blindness, these mice exhibited intact pupillary responses to light that were slower and lower in amplitude compared with normal mice (11). Keeler speculated that retinal ganglion cells or other cell types in the eye might be activated directly (12), but critics argued that *rd/rd* mice were not actually blind and that Keeler's observations could be explained by sparing of visual photoreceptors in the outer retina (13). Criticism of Keeler's work was addressed several decades later when *rd/rd* mice were crossed with *cl* mice, resulting in complete ablation of rods and cones. Pupillary light responses were intact in *rd/rd cl* mice (14), suggesting that a non-rod, non-cone photoreceptor in the mammalian eye was capable of mediating the pupillary light reflex. Similarly, *rd/rd cl* mice exhibited intact light-induced resetting of circadian rhythms and melatonin suppression (15, 16). In parallel, it was found that pupillary responses were preferentially spared in patients with impaired vision caused by mitochondrial disease (17, 18), and some blind patients with no light perception showed intact circadian and melatonin suppression responses to light (19). These studies provided evidence that visual and non-visual light responses were mediated by distinct photoreceptor pathways.

**Figure 1:**
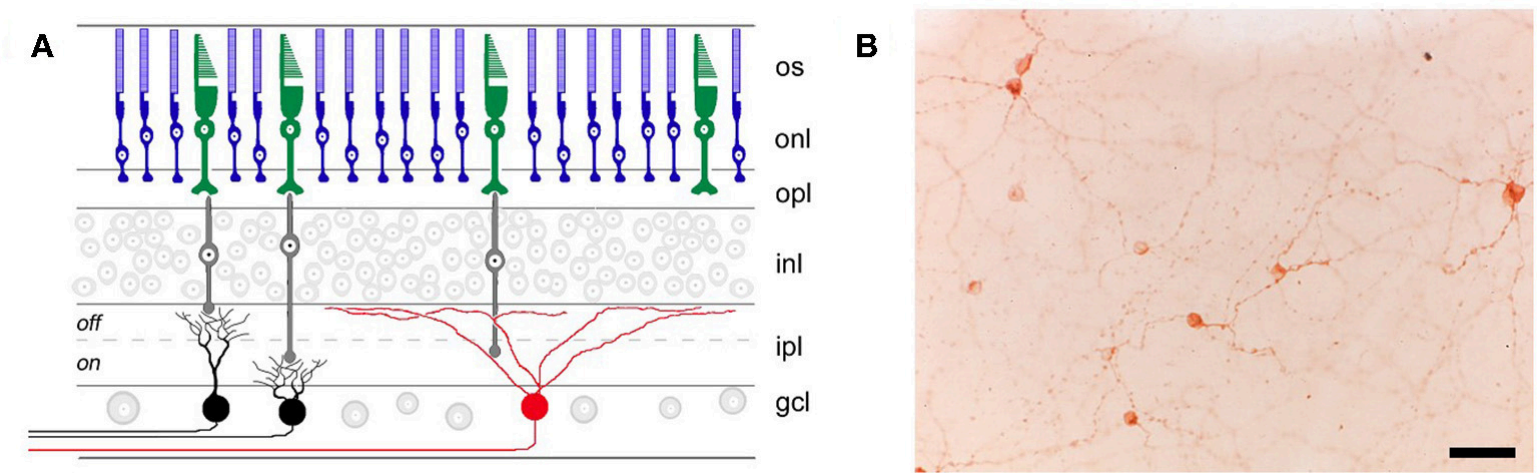
Retinal location of different photoreceptor types. **(A)** Rods (blue) and cones (green) in the outer retina transmit light information via bipolar cells (gray) to retinal ganglion cells (RGCs) in the inner retina. RGCs that are involved in image-forming vision are not directly photosensitive (black), whereas RGCs involved in non-visual light responses (e.g., the pupillary light reflex) contain the photopigment melanopsin (red) and are intrinsically photosensitive. os, outer segments; onl, outer nuclear layer; opl, outer plexiform layer; inl, inner nuclear layer; ipl, inner plexiform layer; gcl, ganglion cell layer. **(B)** Melanopsin-containing RGCs (labeled immunohistochemically in brown) are distributed broadly and in small numbers across the retina, as shown in a flat-mount preparation of a rat retina (scale bar = 50 μm). Panel **(A)** was reproduced with permission from Berson (36). Panel **(B)** is a photomicrograph provided by the corresponding author, JG (Clifford Saper Laboratory, Beth Israel Deaconess Medical Center, Boston, MA).

The discovery of intrinsically photosensitive retinal ganglion cells (ipRGCs) was a turning point in our understanding of the pupillary light reflex and other non-visual light responses. Although ipRGCs can be activated by rod and cone photoreceptors in the outer retina (20, 21), they contain the invertebrate-like opsin melanopsin (*Opn4*) which renders them directly photosensitive (20, 22, 23). Melanopsin cells project to the olivary pretectal nucleus to mediate the pupillary light reflex (22, 24, 25), as well as brain areas involved in circadian rhythms and sleep-wake regulation (22, 24, 26, 27). In mice, there are several types of melanopsin cells (named M1 to M6) that have been identified based on their morphology, central projections, electrophysiological response properties, and their role in different non-visual light responses (28, 29). The M1 ipRGCs in mice that express the transcription factor *Brn3b* project to the olivary pretectal nucleus and are thought to be necessary for the pupillary light reflex (25). Different types of melanopsin cells have also been described in macaques and humans (30, 31), but their role in different non-visual light responses is still under investigation. Melanopsin is required for pupillary light responses in blind mice (32, 33), but visual photoreceptors are capable of mediating the pupillary light reflex in melanopsin knockout mice (34). The pupillary light reflex and other non-visual light responses are abolished only when rod, cone, and melanopsin signaling pathways are disrupted simultaneously (32, 33). Selective ablation of the melanopsin-containing ipRGCs also severely attenuates pupillary responses to light (35), suggesting that most, if not all, light information from outer retinal photoreceptors to the olivary pretectal nucleus is channeled through a few thousand melanopsin cells that are distributed broadly across the retina (Figure 1). It remains possible, however, that conventional RGCs also provide input to the midbrain, either directly or indirectly, to modulate pupillary light responses.

Differences between rod/cone photoreceptors and melanopsin in their anatomic location and response properties has led to renewed interest in using the pupillary light reflex to detect loss of photoreceptor function in retinal and optic nerve diseases. Melanopsin cells are not required for sight in mice (35) and are insufficient to support image-forming vision in blind patients without a functional outer retina (19, 37). Pupillary light responses can be used to estimate damage to the afferent pathway involved in image-forming-vision, however, if the function of ipRGCs and conventional RGCs is similarly impaired by a given disease. Given that rods, cones, and melanopsin play different roles in mediating the pupillary light reflex (38, 39), light stimuli can be designed to stimulate preferentially one or more photoreceptor types, providing a read-out of their function. This serves as the basis for chromatic pupillometry (also termed color pupillometry or selective wavelength pupillometry), which refers to measuring pupillary responses to different wavelengths and intensities of light in order to differentiate rod, cone, and melanopsin-dependent contributions to the pupillary light reflex.

The goal of this article is to review how chromatic pupillometry methods can be used to detect loss of photoreceptor function in retinal and optic nerve diseases. In the first part of this article, we focus on research in humans demonstrating that rod/cone photoreceptors and melanopsin differ in their contributions to the pupillary light reflex. We discuss evidence that the wavelength, irradiance, and duration of a light stimulus can be manipulated to stimulate preferentially rod, cone, or melanopsin-dependent pupillary light responses. In the second part of this article, we review evidence that chromatic pupillometry methods, in particular those that measure pupillary responses to blue light vs. red light, can be used to detect loss of photoreceptor function in diseases that primarily affect either the outer retina or the inner retina. We discuss protocols that use light flashes or short-duration light stimuli to assess the health of rod/cone photoreceptors and ipRGCs (e.g., based on the post-illumination pupillary response), as well as protocols in which pupillary constriction is measured during continuous exposure to light (e.g., stepwise changes in irradiance or ramp-up light exposures). Strengths and limitations of these chromatic pupillometry methods are discussed, with a view toward developing clinical protocols that can be used as part of a routine ophthalmic examination to assess the functional integrity of different photoreceptor types. Finally, we review potential future applications for chromatic pupillometry in screening for retinal diseases, and in monitoring disease progression and/or recovery.

## Photoreceptor Contributions to the Pupillary Light Reflex

Based on electrophysiological studies of ipRGCs in mice and macaques (20, 23), melanopsin-dependent responses differ markedly from those mediated by rod/cone photoreceptors. First, when synaptic transmission from the outer retina is blocked, the action spectrum for the intrinsically-driven (i.e., melanopsin-dependent) light response exhibits peak sensitivity to short-wavelength light in the blue portion of the visual spectrum (λ_max_ ≈ 480 nm). Hence, the spectral maximum for melanopsin differs from human rods (λ_max_ ≈ 505 nm) (40, 41) and short, medium, and long-wavelength cones (42–44). Second, melanopsin-dependent ipRGC responses to light are less sensitive than extrinsically-driven responses mediated by rods and cones. Therefore, the ipRGCs can be activated by outer retinal photoreceptors below the threshold of activation for the intrinsic, melanopsin-dependent light response. Third, melanopsin-dependent light responses of ipRGCs are slower and last longer, relative to rod/cone-dependent responses. The intrinsic response shows a longer response latency following light stimulus onset, and is sustained for as long as the light stimulus is presented. The intrinsic response also extends markedly after light offset unlike rod and cone signaling (20, 21, 23). As reviewed in the following sections, these response characteristics closely match those of the pupillary light reflex in humans, demonstrating complementary roles of outer retinal photoreceptors and melanopsin in mediating pupillary responses to light.

### Melanopsin-Dependent Pupillary Responses Are Sensitive to Short-Wavelength Light

**Figure 2:**
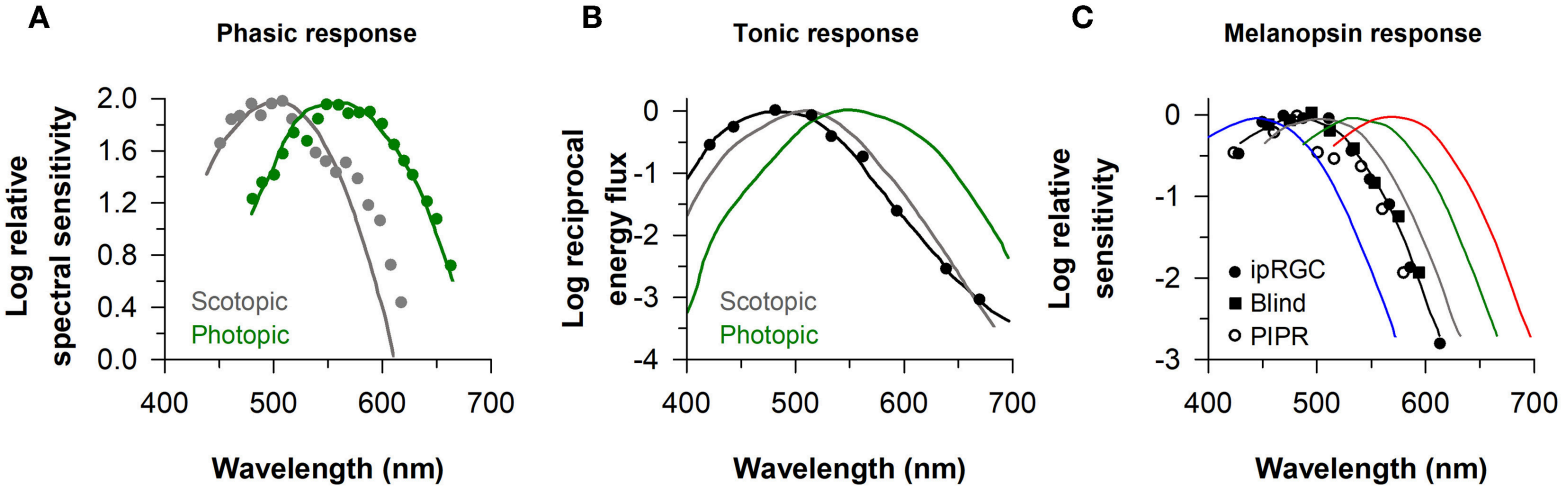
Spectral responses of the pupillary light reflex. **(A)** The spectral sensitivity for pupillary responses to flashes of dim light (gray circles) and bright light on a rod-suppressing blue background (green circles) are shown for representative subjects. Spectral responses for the phasic pupillary light reflex closely resemble scotopic and photopic luminosity functions (gray and green lines, respectively). **(B)** The spectral sensitivity for the tonic pupillary light reflex during continuous exposure to light (black circles) is short-wavelength shifted (peak constriction, ≈ 490 nm) relative to scotopic and photopic luminosity functions (gray and green lines, respectively). **(C)** The spectral sensitivity of melanopsin-dependent responses (peak constriction, ≈ 480 nm) is shown for intrinsically-photosensitive retinal ganglion cells in macaques (ipRGCs, black circles), the pupillary light reflex in a blind individual with no light perception (Blind, black squares), and the sustained post-illumination pupillary light response in individuals with normal vision (PIPR, open circles). The spectral sensitivity of rod and cone photoreceptors is shown for comparison (rods, gray; S-cones, blue; M-cones, green; L-cones, red). Panel **(A)** is redrawn and modified with permission from (2); Panel **(B)** was redrawn and modified with permission from (46); Panel **(C)** was redrawn and modified with permission from (23), with data superimposed from (37) and (47).

The identity of photoreceptors that contribute to the pupillary light reflex has been investigated by measuring the sensitivity of pupillary responses to light as a function of wavelength. In studies that have examined the minimum amount of light energy required to elicit a detectable change in size of the dark-adapted pupil, spectral responses to light flashes closely resemble the scotopic luminosity function (λ_max_ ≈ 500–510) (2, 5, 45) (Figure 2A). If the effects of rod stimulation are masked by providing a background of blue light to render them insensitive, threshold spectral responses to monochromatic light stimuli are higher and closely match the photopic luminosity function (λ_max_ ≈ 555 nm) (2, 45) (Figure 2A). These studies implicate rods and cones in mediating pupillary responses to short-duration light exposures. When the pupil is measured during exposure to continuous dim light (e.g., after 20–30 s of continuous light) below the threshold of color vision, pupillary responses are also matched well by the scotopic luminosity function (3, 4). In the photopic visual range, however, pupillary responses to continuous light are not well matched by either scotopic or photopic luminosity functions (Figure 2B). With the exception of one study (2), pupillary responses during continuous exposure to light were most sensitive to short-wavelength light (λ_max_ ≈ 480–500 nm) (4, 39, 45, 46, 48). These findings are consistent with an important role for melanopsin in mediating the sustained (i.e., tonic) pupillary light reflex. As discussed below, however, detailed analyses of spectral responses suggest that rod and cone photoreceptors contribute substantially during the early part of a continuous light exposure, and outer retinal photoreceptors mediate the tonic pupillary light reflex at low irradiances (38, 39).

The response properties of melanopsin-dependent pupillary light responses can be studied in isolation in blind individuals with complete loss of visual function and degeneration of the outer retina, but with a relatively intact retinal ganglion cell layer (19, 37, 38, 49). In a blind woman with autosomal-dominant cone-rod dystrophy and no detectable rod or cone function (no light perception or electroretinography response, and no outer retina based on fundus photography and ocular coherence tomography), the action spectrum for the pupillary light reflex showed peak sensitivity to 476 nm light (Figure 2C). In a different blind individual with retinitis pigmentosa and no light perception, the spectral response for monochromatic exposures matched for corneal photon density (13 log photons/cm^2^/s) exhibited peak sensitivity to 490 nm light (38). These results are consistent with action spectra for the pupillary light reflex in *rd/rd cl* mice with complete degeneration of the outer retina (λ_max_ ≈ 479 nm) (14), and in macaques with synaptic blockade of signals from the outer retina (λ_max_ ≈ 479 nm) (47). Moreover, spectral responses for the pupillary light reflex in blind individuals are similar to electrophysiological responses of ipRGCs of mice and macaques with blocked synaptic transmission from rods and cones (λ_max_ ≈ 484 and 482 nm, respectively) (20, 23) (Figure 2C). In humans with normal vision, the action spectrum for the post-illumination pupillary response (i.e., sustained pupillary constriction after light exposure offset) also exhibits peak sensitivity to about 482 nm light (Figure 2C), suggesting that this response is driven predominantly by melanopsin (47, 50). Together, these findings in humans show that melanopsin-dependent pupillary responses are sensitive to short-wavelength blue light (i.e., λ_max_ ≈ 480 nm).

### Melanopsin-Dependent Pupillary Responses Are Less Sensitive to Light Than Rods and Cones

The relative sensitivity of rod, cone, and melanopsin-dependent ipRGC responses has been characterized *in vitro* in the retinae of macaques, which have trichromatic vision similar to humans (23). Following dark adaptation, rod-driven responses in ipRGCs are highly sensitive and can respond to light stimuli as low as 6–7 log quanta/cm^2^/s, which is 4–5 log units below the threshold for (L + M) cone-mediated responses. By comparison, melanopsin-driven responses are about a log unit less sensitive than cones, with a threshold of activation of about 11–12 log quanta/cm^2^/s (23). As reviewed in the previous section, rods mediate the pupillary light reflex at light intensities below the threshold of color discrimination, and cones contribute to phasic pupillary light responses in the photopic visual range. Decades before melanopsin was discovered, there was evidence that neither rods nor cones were necessary for pupillary constriction responses to bright light. In achromats without cone photoreceptor function, it was shown that the pupils could respond to light in a dose-dependent manner well beyond the point of rod saturation (51). After light adaptation, achromats also exhibited short-wavelength sensitivity (λ_max_ ≈ 490 nm) to flashes of light within the photopic visual range (7), hence deviating substantially from responses in normally-sighted individuals (λ_max_ ≈ 555 nm) (2, 45). In a patient with congenital stationary night blindness (Oguchi disease) with total loss of rod function, the sustained pupillary light reflex was severely impaired at low-to-moderate light intensities, but appeared relatively normal during exposure to bright light (6). With the benefit of hindsight, intact pupillary responses to high-irradiance light stimuli in patients with achromatopsia and Oguchi disease were likely due to stimulation of melanopsin.

**Figure 3:**
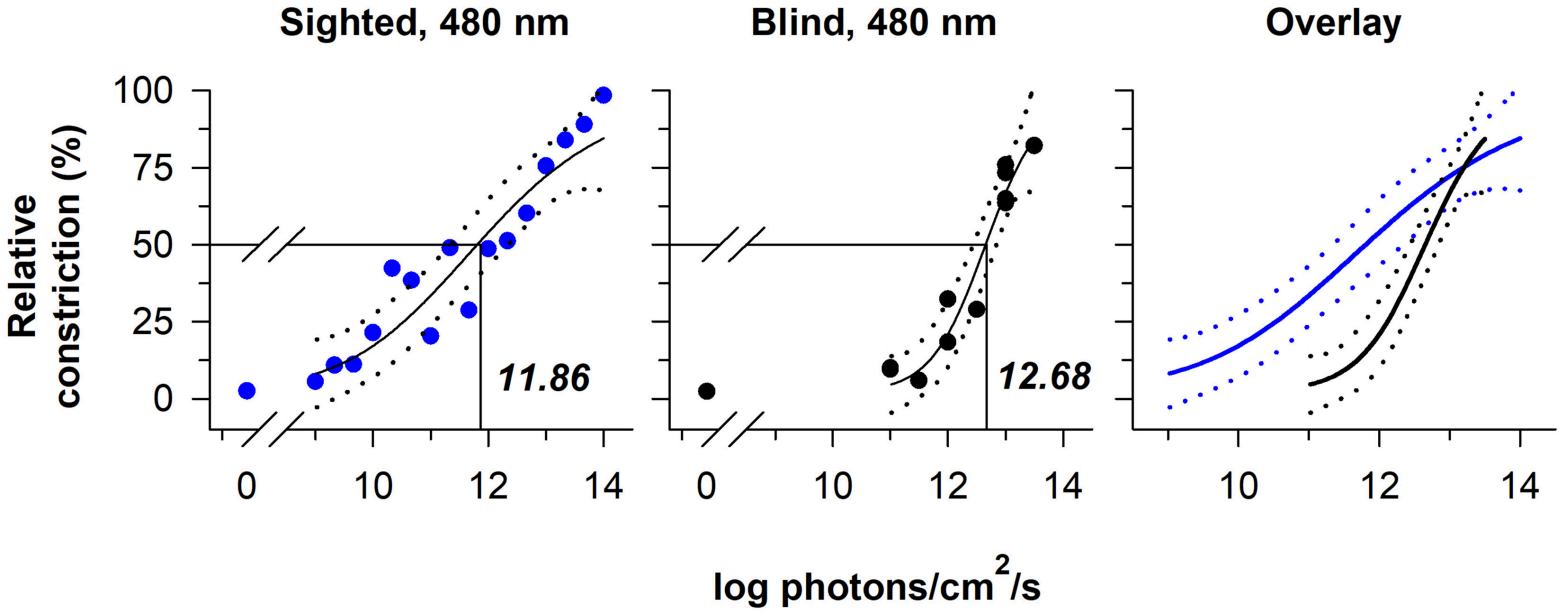
The contribution of rod-cone photoreceptors and melanopsin to pupillary light responses is irradiance-dependent. Irradiance-response curves for the pupillary light reflex are shown for a continuously presented blue-light stimulus (480 nm; pupil measured from 90 to 120 s after light onset) in individuals with normal vision (blue circles, left panel) and in a blind individual with no light perception (black circles, middle panel). An overlay of the irradiance-response curves (right panel) shows reduced pupillary responses to lower-irradiance exposures in the blind individual, whereas the melanopsin-dependent response at the highest irradiances tested was comparable to sighted individuals. In each plot, the best-fit regression line is shown with 95% CIs (solid and dotted lines, respectively). Drop lines indicate the irradiance corresponding to a half-maximal pupillary constriction response. Data are replotted with permission from (38).

The relative sensitivity of melanopsin-dependent pupillary light responses has been studied in a totally-visually blind individual with intact non-visual photoreception (37, 38). Based on irradiance-response curves to 480 nm light, pupillary constriction responses in the blind individual were preserved at higher irradiances, i.e., ≥13 log photons/cm^2^/s measured at the cornea, but were severely attenuated compared with sighted individuals across most of the photopic range of vision (Figure 3). These results are consistent with findings in *rd/rd* mice and *rd/rd cl* mice in which pupillary light responses were reduced except at the highest irradiances tested (>13 log quanta/cm^2^/s) (33, 34). In contrast, *Opn4* null mice show normal pupillary light responses at lower irradiances, but reduced responses to higher-intensity light stimuli (>13 log quanta/cm^2^/s) (33, 34). The threshold for pupillary constriction in the blind individual (≈11–12 log photons/cm^2^/s) was also similar to results reported for pupillary light responses in mice that lack rod and cone function (34, 52), and in macaques in which rod and cone signaling was pharmacologically blocked (47). Together, these findings demonstrate that outer retinal photoreceptors are required for normal pupillary responses at low-to-moderate intensities of light, whereas melanopsin alone is sufficient to drive a normal pupillary light reflex in the presence of a bright, continuous light stimulus (especially blue light). Even in normally-sighted individuals, pupillary responses to bright light (using a criterion of a 75% maximum constriction response) are consistent with the spectral sensitivity of melanopsin, suggesting that melanopsin dominates the pupillary light reflex at high irradiances (39).

### Melanopsin-Dependent Pupillary Responses to Light Are Slow and Sustained

The pupillary light reflex is often described as having phasic and tonic components. The phasic component refers to the transient, high-amplitude response that occurs in response to a light flash or at the beginning of a continuous light stimulus. By comparison, the tonic component refers to the sustained pupillary response that occurs during continuous exposure to light. To a large degree, phasic and tonic components of the pupillary light reflex reflect the contribution of rod/cone photoreceptors and melanopsin to ipRGC responses (20, 23). Rods and cones contribute strongly to ipRGC responses and pupillary constriction at the beginning of light exposure (e.g., over seconds to minutes), but their contribution declines substantially over time (23, 39, 53). After the phasic component of the pupillary light reflex, rods and melanopsin appear to contribute to sustained ipRGC and pupillary light responses (21, 23, 54). Consequently, there is a short-wavelength shift in spectral sensitivity over time during a continuous light stimulus, with responses to higher-irradiance light dominated by melanopsin (38, 39, 48). Studies using the silent substitution method have provided additional evidence that melanopsin contributes to sustained pupillary constriction in the photopic visual range, with lesser contributions from rods and/or (L + M) cones (55, 56). Consistent with a role for outer retinal photoreceptors in mediating the tonic component of the pupillary light reflex, sustained pupillary constriction can be driven by long-wavelength red light (631 nm; for at least 50 min) outside the range of sensitivity of melanopsin-dependent ipRGC responses (57).

**Figure 4:**
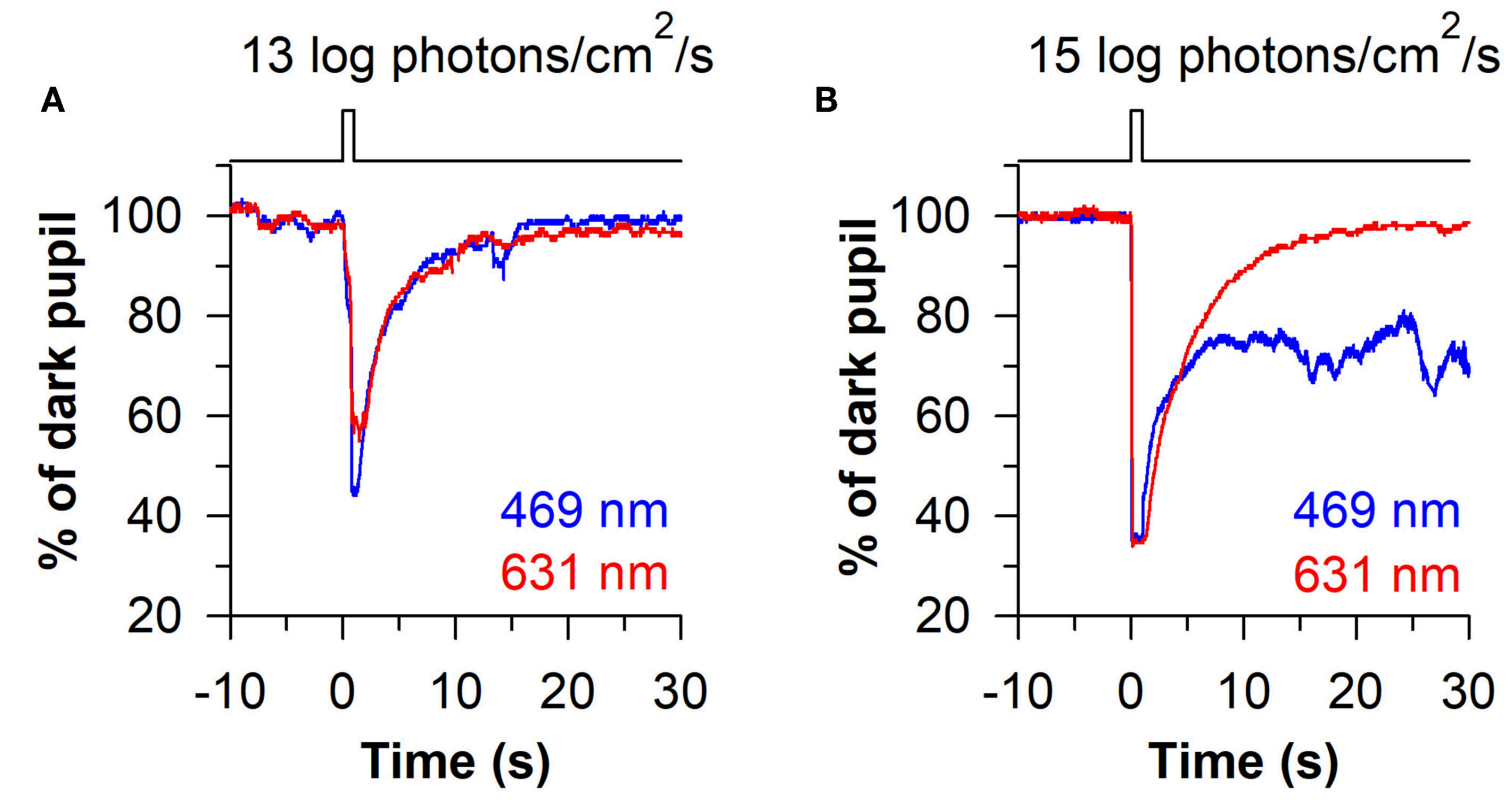
Phasic and sustained pupillary responses to 1-s flashes of blue or red light. **(A)** Pupillary responses are shown for a representative individual exposed to a moderately-bright 1-s flash of either blue light or red light (469 or 631 nm; 13 log photons/cm^2^/s). The pupil showed a transient high-amplitude constriction response after light onset, with fast re-dilation of the pupil after light offset. **(B)** In response to a high-intensity 1-s flash of light (15 log photons/cm^2^/s), the transient pupillary constriction response was followed by a prolonged post-illumination pupillary light response (PIPR) to the blue light stimulus, but not the red light stimulus. The sustained PIPR is thought to be driven by slow-deactivation of melanopsin after light offset. The figure is replotted with permission from (58).

The time-course of pupillary re-dilation after light stimulus offset also appears to have phasic and tonic components that are differentially influenced by rod/cone photoreceptors and melanopsin (38, 39). The pupil typically re-dilates rapidly (i.e., over the course of several seconds) toward its dark-adapted size after exposure to a light stimulus of low-to-moderate intensity (Figure 4A). In contrast, the pupil can show a sustained constriction response (i.e., over tens of seconds) after exposure to a high-intensity light stimulus (Figure 4B). The phasic component of pupillary re-dilation is absent or markedly delayed in blind humans and mice lacking a functional outer retina (38, 59), demonstrating that rods and cones contribute to the fast pupillary response after light stimulus offset. As shown in humans and in macaques, the sustained post-illumination pupillary response (PIPR) is driven predominantly by melanopsin and is sensitive to short-wavelength blue light (47). Consequently, a strong PIPR can be driven after the offset of a bright-blue light stimulus, whereas there is little or no PIPR after a red-light stimulus (Figure 4B). Notably, the PIPR in visually-normal individuals is much greater when the light stimulus is presented to the dark-adapted eye compared with an adapting background field, indicating that pre-exposure light conditions modulate the strength of the PIPR (60).

**Figure 5:**
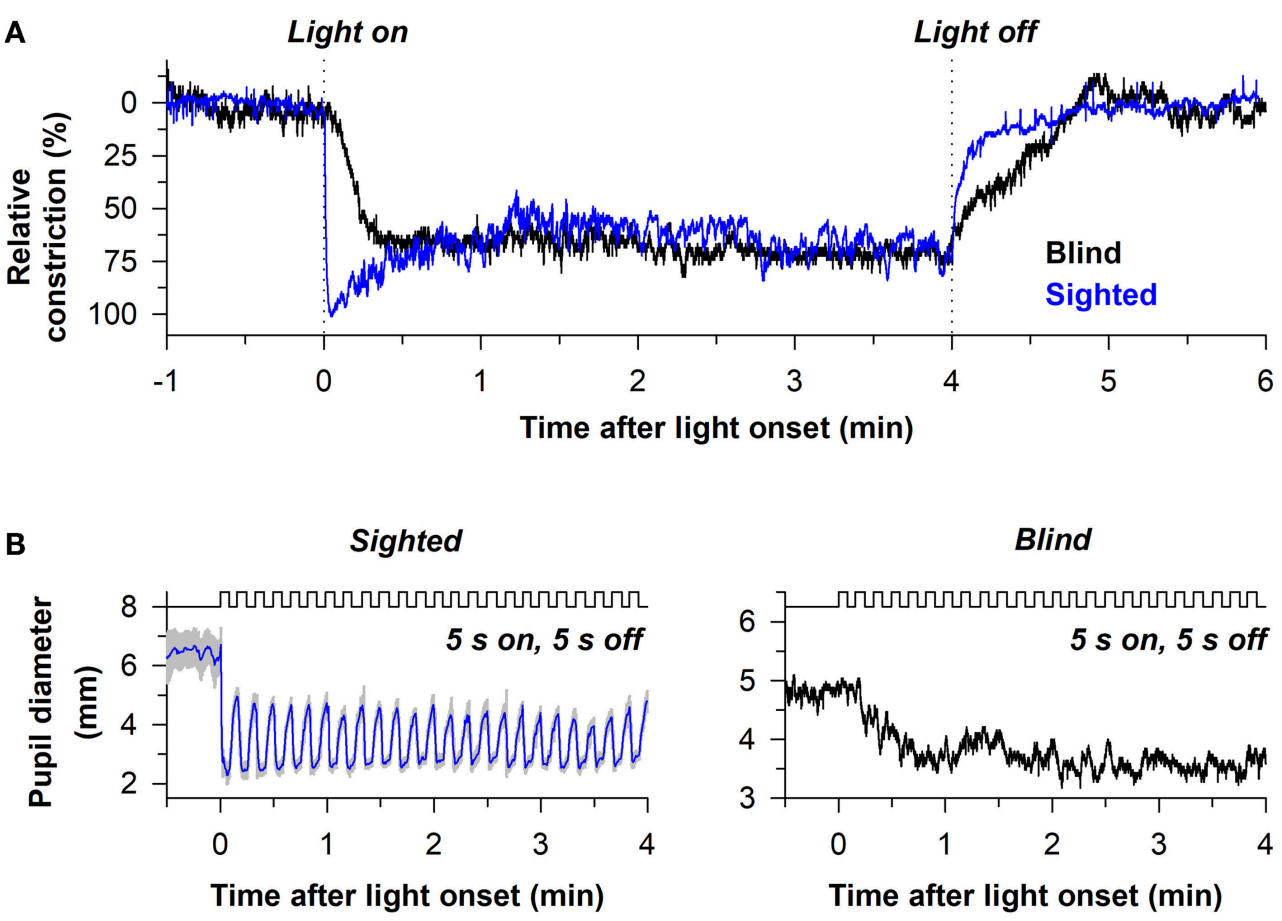
Melanopsin-dependent pupillary responses are slower than rod/cone-dependent responses. **(A)** Representative pupillary light responses are shown for a sighted individual and a blind individual without rod and cone function. The pupil in the blind individual responded slowly after light onset and light offset, indicating that outer retinal photoreceptors are necessary for the phasic component of the pupillary light reflex. **(B)** In sighted individuals, the pupil could track an intermittent light stimulus (480 nm, 13 log photons/cm^2^/s) with alternating periods of light and darkness (5 s of light, 5 s of darkness). By comparison, the pupil in the blind individual was unable to track the intermittent stimulus. Rather, the melanopsin-dependent pupillary response increased across several light pulses until reaching a steady response. Data are replotted with permission from (38).

The sluggish response properties of melanopsin have been studied in detail in a blind individual without a functional outer retina (Figure 5A) (38). In the blind person, the pupil responded slowly to light stimulus onset, often taking several seconds to show a detectable response, with a response latency that decreased linearly with increasing light intensity. By comparison, the rapid pupillary light response in sighted individuals masked the slow contribution of melanopsin to the pupillary light reflex at the start of a continuous light exposure. After the pupil reached its minimal size in the blind individual, the melanopsin-dependent pupillary response was sustained, similar to the response in sighted individuals for a high-irradiance light stimulus. After light stimulus offset, however, the pupil in the blind participant re-dilated slowly compared with sighted individuals (Figure 5A), suggesting that the melanopsin-dependent PIPR was unmasked or enhanced in the absence of rod and cone function (61–63). Due to the slow time course of pupillary light responses in the blind individual, his pupil was unable to track an intermittent light stimulus (5 s on, 5 s off), whereas pupillary constriction and dilation responses were time-locked to each light and dark pulse in sighted individuals (Figure 5B). These findings suggest that rods and cones are superior to melanopsin at encoding rapid changes in light intensity (i.e., abrupt changes in light). Hence, rod/cone photoreceptors likely dominate phasic pupillary responses to the onset and offset of a light stimulus, whereas melanopsin can track low-contrast modulation of light intensity (64).

### Summary of Photoreceptor Contributions to the Pupillary Light Reflex

Outer retinal photoreceptors and melanopsin contribute differentially to the pupillary light reflex. Melanopsin-dependent pupillary responses are short-wavelength sensitive compared with rod- and cone-mediated responses and have a higher threshold of activation. Additionally, rods and cones dominate the phasic component of pupillary responses after light stimulus onset and offset. In contrast, melanopsin-dependent responses are much slower and sustained, and dominate the tonic component of the pupillary light reflex during exposure to high-irradiance, continuous light stimuli. Rods can mediate the tonic pupillary light reflex at low-to-moderate light intensities, whereas the role of cones is uncertain. Following the offset of a high-intensity short-wavelength light stimulus, melanopsin dominates the slower component of the PIPR, resulting in a slower re-dilation of the pupil toward its dark-adapted size. These findings demonstrate that responses of rod/cone photoreceptors and melanopsin can be assessed differentially using the pupillary light reflex. This has given rise to chromatic pupillometry, in which the functional integrity of photoreceptors in the outer retina and inner retina can be examined using light stimuli that target rods, cones, or melanopsin.

## Chromatic Pupillometry Methods for Assessing Retinal and Optic Nerve Diseases

Chromatic pupillometry methods exploit differences in response characteristics of rod/cone photoreceptors and melanopsin to assess damage to the outer retina and inner retina, respectively. Over the past decade, several types of light exposure protocols have been developed to assess the functional integrity of rods, cones, and ipRGCs. Most of these studies have compared pupillary responses to blue light and red light using light-emitting diodes, in order to isolate as best as possible the function of outer retinal photoreceptors vs. ipRGCs. Rod-mediated pupillary responses have the lowest threshold of activation, and hence low-irradiance blue light stimuli in the scotopic visual range can be used to test for rod function (e.g., dim light flashes after dark adaptation). Cone-mediated pupillary responses are less sensitive to light than rods and are preferentially sensitive to longer-wavelength light. Therefore, red light stimuli in the photopic visual range can be used to test for cone function (e.g., red light flashes after light adaptation). Melanopsin-dependent pupillary responses are the least sensitive to light and are preferentially sensitive to short-wavelength blue light. As such, high-irradiance blue light stimuli can be used to test for ipRGC function. Chromatic pupillometry protocols can be categorized broadly as those using short-duration light stimuli to assess photoreceptor health (e.g., light flashes or pulses), or those using continuously presented light stimuli (e.g., >30 s). Here, we review evidence that either of these approaches can be used to detect inner vs. outer retinal damage.

### Chromatic Pupillometry Methods That use Short-Duration Light Stimuli

#### Assessing Photoreceptor Function Using 1-s Light Flashes

An important goal of chromatic pupillometry is to develop a standardized clinical protocol for assessing the health of retinal photoreceptors. Using a Ganzfeld system with full-field illumination of the eye (with the other eye covered with a patch), it was shown that a series of 1-s light exposures (470 nm blue light or 640 nm red light) could be used to assess rod, cone, and melanopsin contributions to the pupillary light reflex (60). Rod function was tested using a dim blue light stimulus after 10 min of dark adaptation (−3 or −2 log cd/m^2^), cone function was tested using a bright red light stimulus under light adaptation with a rod-suppressing blue background (2.6 log cd/m^2^ of red light on a background of 0.78 cd/m^2^ of blue light), and melanopsin function was tested using a bright blue light stimulus after dark adaptation (2.6 log cd/m^2^) and measured using the PIPR. Pupillary responses under these lighting conditions were then compared between normally-sighted individuals and patients with either retinitis pigmentosa (*n* = 5) or Leber congenital amaurosis (LCA, *n* = 3). Consistent with loss of rod function and a higher threshold of activation for the pupillary light reflex (65, 66), patients with outer retinal disease showed weak or absent pupillary responses to the rod-weighted dim blue light stimulus, as well as attenuated responses to the cone-weighted red light stimulus (60). In contrast, the PIPR for the melanopsin-weighted stimulus appeared normal in patients with retinitis pigmentosa or LCA after dark adaptation. Interestingly, the PIPR was prolonged in patients with outer retinal disease compared with controls when bright blue light was presented on a blue background (2.6 log cd/m^2^ of blue light on a background of 0.78 cd/m^2^ of blue light), suggesting that light adaptation did not suppress melanopsin-dependent responses to the same extent as seen in healthy participants.

**Figure 6:**
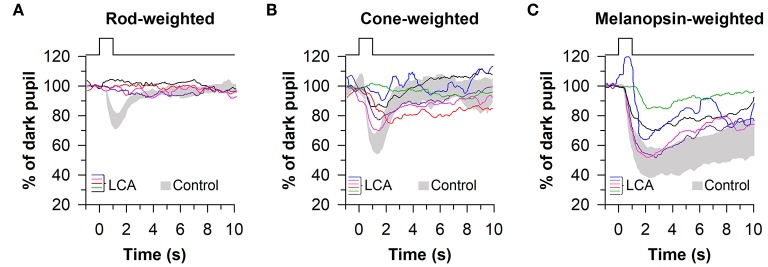
Protocol for assessing photoreceptor health using 1-s light flashes. Pupillary responses were assessed in patients with Leber congenital amaurosis (LCA), using light stimuli designed to stimulate preferentially rods, cones, or melanopsin. **(A)** Pupillary responses to the rod-weighted light stimulus (465 nm, −3 log cd/m^2^ after 10 min of dark adaptation) were non-recordable in LCA patients. **(B)** Responses to the cone-weighted light stimulus (642 nm, 1 log cd/m^2^ on a blue background of 0.78 log cd/m^2^) were reduced in LCA patients compared with healthy controls. **(C)** The post-illumination pupillary response to the melanopsin-weighted stimulus (2.6 log cd/m^2^) was intact in LCA patients but the amplitude was reduced. Gray traces show the range of pupillary responses in the control group. The figure is redrawn and modified with permission from (67).

These findings were confirmed and extended using a similar light exposure protocol in patients with *CEP290*-associated LCA (*n* = 6), demonstrating reduced pupillary responses to rod- and cone-weighted light stimuli and an intact PIPR to the melanopsin-weighted stimulus after dark adaptation (Figures 6A–C) (67). Comparable results were obtained in another study in which pupillary responses were assessed in patients with LCA or early-onset severe retinal dystrophy (*n* = 6) caused by *RPE65* mutations (68), except that the PIPR measured after light offset for the bright blue light stimulus (percentage of constriction after 30 s) was greater in patients compared with controls. The ability of this protocol to detect selective loss of cone function has also been demonstrated in a pair of patients with mutations in the *CNGB3* gene, resulting in achromatopsia. In these patients, pupillary responses to rod- and melanopsin-weighted light stimuli were in the normal range, whereas pupillary responses were severely blunted for the cone-weighted light stimulus (68). In contrast, in patients with idiopathic intracranial hypertension (*n* = 13), which can result in ischemia of the optic nerve, pupillary responses were reduced for rod-, and melanopsin-weighted light stimuli, demonstrating reduced ipRGC transmission to the midbrain (69). In another study, patients with moderate-to-severe non-proliferative diabetic retinopathy (NPDR) showed normal pupillary responses to the rod-weighted light stimulus and reduced responses to the cone-weighted light stimulus (70). Patients with mild or moderate-to-severe NPDR also exhibited an attenuated PIPR in the melanopsin-weighted light condition, suggesting damage to both the outer and inner retina.

Pupillometry protocols have also been developed to assess the irradiance-dependent effects of blue and red light stimuli (1-s flashes) on the pupillary light reflex in patients with retinal or optic nerve disease. In such protocols, irradiance-response curves are constructed by exposing participants to a sequence of 1-s light flashes that increase in intensity over time, with each stimulus preceded by a period of darkness (or on a background of continuous light) to allow the pupil to re-dilate before the next stimulus is administered. Loss of rod or cone function can be inferred by reduced sensitivity to blue and red light stimuli compared with individuals with normal vision (i.e., a rightward shift in the irradiance-response curve), whereas impairment of the melanopsin-dependent ipRGC response can be assessed by the PIPR after exposure to high-intensity blue light. This method has been tested in patients with LCA (*n* = 4) using a Ganzfeld system, in which pupillary responses (i.e., peak pupillary constriction, normalized to the baseline pupil) to 1-s red light (640 nm) and blue light (467 nm) stimuli were measured over a 6 log unit range of intensities matched for photopic luminance (−4.0 to 2.0 log cd/m^2^, increased in 0.5 log steps) (71). Patients with LCA exhibited decreased sensitivity to blue light with severely reduced pupillary responses for dim light stimuli (<-1.0 log cd/m^2^), whereas pupillary responses were in the normal range for bright red light stimuli. These findings are consistent with degeneration of rods and loss of scotopic visual function, with sparing of cone-mediated responses. The PIPR for bright blue light (2.6 log cd/m^2^) was also in the normal range in patients with LCA, which is consistent with intact retinal ganglion cell function. Comparable results were obtained in patients with autosomal dominant retinitis pigmentosa caused by a mutation in the *NR2E3* gene (*n* = 9; 1-s exposures blue light; −6.0 to 1 log cd/m^2^ in 0.5 log unit steps), in which rod-dependent pupillary responses to blue light exhibited reduced sensitivity compared with healthy controls (72). Using a protocol in which red light flashes were presented on a background of rod-suppressing blue light (−1.0 to 1.5 log cd/m^2^ of red light after 3 min of light adaptation to 0.78 log cd/m^2^ of blue light), cone-weighted responses were in the normal range in patients with retinitis pigmentosa. The PIPR after exposure to bright blue light (2.6 log cd/m^2^) was intact but marginally reduced in patients, indicating that function of the inner retina was largely preserved. Similar results were observed in patients with retinal dystrophy caused by enhanced S-cone syndrome (*n* = 4), in which autosomal recessive mutations in the *NR2E3* gene result in an overabundance of S-cones and reduced function of rods (73). In these patients, rod-dependent pupillary responses were undetectable and cone-dependent responses to red light were slightly attenuated, whereas pupillary responses to the melanopsin-weighted stimulus were in the normal range.

Irradiance-dependent responses to 1-s light flashes have also been examined in patients affected by various types of optic neuropathies. In patients with mild-to-moderate visual dysfunction due to hereditary optic neuropathy (HON; *n* = 8), dose-response curves to rod-weighted blue light stimuli (463 nm, −4.0 to −1.0 log cd/m^2^ in 0.5 log unit steps with intervening dark periods) and cone-weighted red light stimuli (635 nm, 1.0 to 2.5 log cd/m^2^ in 0.5 log unit steps on a background of 92 lux of light) were comparable to responses observed in healthy controls (74). The PIPR after exposure to bright blue light (2.3 log cd/m^2^) in these patients was also in the normal range, indicating preserved melanopsin-dependent ipRGC function. In another study, pupillary responses were examined in 10 patients with Leber HON (LHON), having severe visual field loss and marked thinning of the retinal nerve fiber layer (RNFL), quantified with optical coherence tomography (OCT) (75). In these LHON patients, the phasic pupillary responses to blue light and red light stimuli (0, 1, 2, and 2.4 log cd/m^2^) were reduced relative to healthy controls, but there was substantial overlap in responses between groups. The PIPR after exposure to bright blue light was also only modestly reduced in patients with LHON, suggesting that ipRGC function was preferentially spared relative to conventional retinal ganglion cells involved in image-forming vision. Similarly, in another study that measured the PIPR after a 1-s flash of bright blue light, the post-stimulus pupil size (at 6 s after light offset) was not different between HON patients (*n* = 11) and healthy controls (76).

Several studies have suggested that ipRGCs are resistant to neurodegeneration in mitochondrial optic neuropathies (i.e., LHON and autosomal dominant optic atrophy) (77–79), but they are vulnerable in other, more common types of optic neuropathies (i.e., patients with ischemic, inflammatory, or glaucomatous optic neuropathies). In a series of patients with anterior ischemic optic neuropathy (AION; *n* = 18) the sensitivity of pupillary responses to blue light (464 nm, −4.0 to −1.0 cd/m^2^ in 0.5 log unit steps with intervening dark periods) and red light (635 nm, 1.0 to 2.5 log cd/m^2^ in 0.5 log unit steps under light adaptation to 90 cd/m^2^) was in the normal range, but the PIPR after exposure to bright blue light was impaired in eyes affected by AION, compared with contralaterally unaffected eyes and healthy control eyes (80). These findings indicate that ipRGCs are damaged following ischemic injury to the optic nerve. Several studies have also shown that the PIPR to a 1-s flash of short-wavelength light is impaired in patients with glaucomatous optic neuropathy compared with healthy controls (76, 81–84). Moreover, in glaucoma patients (*n* = 38), the magnitude of the PIPR (normalized pupil size measured 6 s after light offset) after exposure to a bright blue light stimulus (470 nm, 2.4 log cd/m^2^) correlated with the magnitude of visual field loss assessed by standard automated perimetry (SAP), and RNFL thickness assessed by OCT (81). Similar results were obtained in another study in patients with glaucoma (*n* = 46) in which the consensual pupillary light reflex was assessed after monocular exposure to either a full-field or superonasal-quadrant light stimulus (464 nm, 2.9 log cd/m^2^) (84). The PIPR amplitude (6 s after light offset) in glaucomatous eyes was associated with visual field deficits and RNFL thinning, suggesting that reduced melanopsin-dependent pupillary responses might be used as a proxy to estimate the loss of conventional retinal ganglion cells involved in image-forming vision.

#### Assessing ipRGC Function Using the PIPR After a 10-Second or 20-Second Light Stimulus

**Figure 7:**
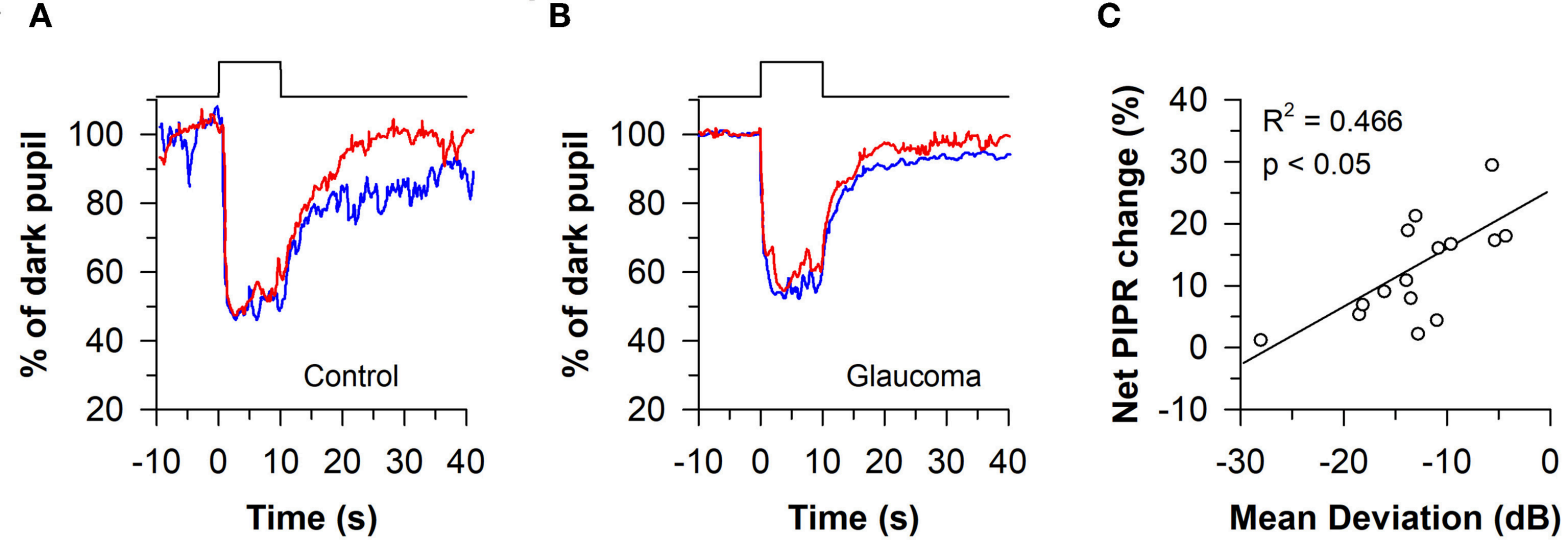
Protocol for assessing melanopsin-dependent pupillary responses using the post-illumination pupillary light response (PIPR). The PIPR was assessed after exposure to 10 s of bright blue light or red light (488 or 610 nm; 14.2 log quanta/cm^2^/s). **(A)** In individuals with normal vision, the PIPR to blue light was greater than the PIPR to red light for at least 30 s after light offset. **(B)** In patients with glaucoma, the PIPR to blue light was reduced relative to red light, indicating reduced light transmission from melanopsin-containing retinal ganglion cells. **(C)** In glaucomatous eyes, the net PIPR change (the difference in the PIPR to blue light vs. red light, adjusted for baseline pupil size) correlated with visual field loss assessed by Humphrey visual field mean deviation. Panels **(A,B)** are redrawn with permission from (87). Panel **(C)** is redrawn with permission from (86).

The melanopsin-dependent PIPR was first demonstrated in humans and macaques using a 10-s light stimulus protocol (47). Prior work had shown that macaque ipRGCs studied *in vitro* continued to fire long after the offset of a 10-s light exposure (23). Hence, subsequent clinical studies have also investigated the PIPR using a 10-s light stimulus, with average pupil size measured from 10 to 40 s after light offset. Using this approach, the consensual PIPR was characterized in healthy individuals (*n* = 45) after exposing the other eye to a bright blue light or red light stimulus, centered on the pupil in Maxwellian view (470 nm vs. 623 nm; retinal irradiance of 13 log quanta/cm^2^/s with a fully-dilated pupil using a mydriatic agent) (85). Although there were substantial individual differences in the magnitude of pupillary constriction, all participants exhibited a sustained PIPR based on the change in pupil size after exposure to blue light relative to red light, adjusted for the percentage change in pupil size relative to the baseline pupil diameter (i.e., the net PIPR change, determined by subtracting the blue PIPR percentage value from the red PIPR percentage value). Similar methods have been used to examine ipRGC function in patients with glaucoma (86, 87). After exposure to 10 s of bright light (488 vs. 610 nm; corneal irradiance, 14.2 log quanta/cm^2^/s), the net PIPR to blue light vs. red light was reduced in patients with advanced glaucoma (*n* = 11) compared with healthy controls (Figures 7A,B), but there was no difference in the PIPR between patients with early glaucoma (*n* = 14) and healthy controls (87). In another study, the net PIPR change in glaucoma patients (*n* = 16) after exposure to bright light (470 vs. 623 nm; retinal irradiance of 13 log quanta/cm^2^/s) was linearly correlated with the magnitude of visual field loss (Figure 7C), demonstrating that ipRGC dysfunction in glaucoma is associated with disease severity (86). In patients with type 2 diabetes without diabetic retinopathy (*n* = 7), the PIPR to bright light (488 vs. 610 nm; corneal irradiance, 14.2 log quanta/cm^2^/s) was also reduced and associated with the duration of diabetes (88), suggesting that ipRGC dysfunction may occur in diabetes prior to onset of visual loss. Similarly, in patients with age-related macular degeneration (AMD; *n* = 2), the PIPR to bright light (464 vs. 635 nm; corneal irradiance, 15 log photons/cm^2^/s) was reduced in both early and advanced, neovascular AMD compared with controls (89).

In other studies, the PIPR to a bright 20-s light stimulus has been used to examine ipRGC function. In a patient with LHON with unilateral visual loss, sustained pupillary constriction after light offset (460 vs. 660 nm; 2.0 or 2.5 log cd/m^2^) did not differ between the affected eye and the healthy eye, suggesting that ipRGC function was resistant to effects of the disease (90). In contrast, in patients with unilateral AION (*n* = 10), pupillary responses of the affected eye were reduced compared with the contralateral, non-affected eye, both during and after exposure to 20-s of bright light (470 vs. 660 nm; 2.5 log cd/m^2^) (91). In patients with choroideremia (*n* = 18), which is characterized by progressive degeneration of the outer retina, the peak pupillary constriction response was also reduced during exposure to bright blue light or red light (463 vs. 643 nm; 100 lux), but the PIPR was intact and lasted longer compared with healthy individuals (92). Together, these results are consistent with studies that used 1-s light flashes (see previous section), showing that the PIPR is reduced in diseases that result in damage to ipRGCs, but remains largely intact in diseases that primarily affect the outer retina.

### Chromatic Pupillometry Methods That use Continuously Presented Light

#### Assessing Photoreceptor Function Using Stepwise Increases in Light Intensity

The pupillary light reflex shows a phasic (i.e., transient) constriction response at the beginning of a continuous light stimulus, followed by a tonic (i.e., sustained) response that is lower in amplitude. Hence, deficits in outer retinal function can be assessed by the phasic response to a step of blue or red light, and deficits in inner retinal function can be assessed by the tonic pupillary response and the PIPR to high-intensity blue light. These response characteristics can be investigated using protocols in which the light intensity is increased stepwise over time. Using a Ganzfeld system to administer light to the eye continuously under mesopic conditions (with the non-stimulated eye covered), the direct pupillary response to a sequence of increasing blue or red light stimuli was measured over a 2 log unit range, with each light step presented for 13 s and matched for photopic luminance (467 and 640 nm; 0, 1, and 2 log cd/m^2^; Figure 7) (93). The transient pupillary response was defined as the maximum constriction response measured within 180–500 ms after light onset, and the sustained pupillary response was defined as pupillary constriction at the 13th second of stimulation for each light step (with each measure adjusted for pre-stimulus pupil size). After characterizing pupillary responses in healthy individuals, this protocol was tested in a patient with retinitis pigmentosa with loss of rod function and reduced cone function based on electroretinography, a patient with achromatopsia in whom cone function was disrupted due to a mutation in the *CNGA3* gene, and a patient with unilateral AION with severe visual loss in the affected eye (93). The patient with retinitis pigmentosa showed deficits in phasic and tonic pupillary responses to the rod-weighted dim blue light stimulus, whereas the melanopsin-weighted response to bright blue light was in the normal range. Responses to moderate-to-bright red light were also attenuated, which is consistent with reduced cone function. In the achromat, pupillary responses to blue light were comparable to healthy controls, suggesting normal function of rods and melanopsin, whereas the cone-weighted response to red light was on the lower end of the normal range, perhaps due to activation of rods (see below). In the AION patient, pupillary responses in the affected eye were markedly reduced for all light stimuli, indicating reduced function of ipRGCs and their input from the outer retina.

This protocol was subsequently evaluated in a group of patients with retinitis pigmentosa (*n* = 32) (62), in whom the transient pupillary response to dim blue light (467 nm; 0 log cd/m^2^) was defined as the rod-weighted light response, and the transient pupillary response to bright red light (640 nm; 2 log cd/m^2^) was defined as the cone-weighted response. The melanopsin-dependent pupillary response was assessed by sustained pupillary constriction at the end of the bright blue light stimulus (467 nm; 2 log cd/m^2^). Under these testing conditions, patients with a non-recordable or abnormal scotopic/photopic electroretinogram showed reduced transient pupillary responses to rod- and cone-weighted light stimuli, as well as a reduced tonic response to the melanopsin-weighted light stimulus. However, patients with retinitis pigmentosa showed a slower and more sustained PIPR after exposure to the melanopsin-weighted stimulus, as compared with patients with normal vision. Consistent with these findings, another study that used the same protocol found that patients with mutations in the *RPE65* gene (*n* = 11) exhibited reduced transient pupillary responses to rod- and cone-weighted light stimuli, a small reduction in sustained pupillary constriction during exposure to the melanopsin-weighted stimulus, and a prolonged PIPR after light offset compared with healthy controls (68). In a pair of achromats, pupillary responses were in the normal range for the rod-weighted stimulus, but were attenuated for the cone-weighted stimulus. The finding that achromats could still respond to red light is likely explained by activation of rods by the cone-weighted stimulus because total loss of cone function was confirmed in other experiments in the same individuals (68).

**Figure 8:**
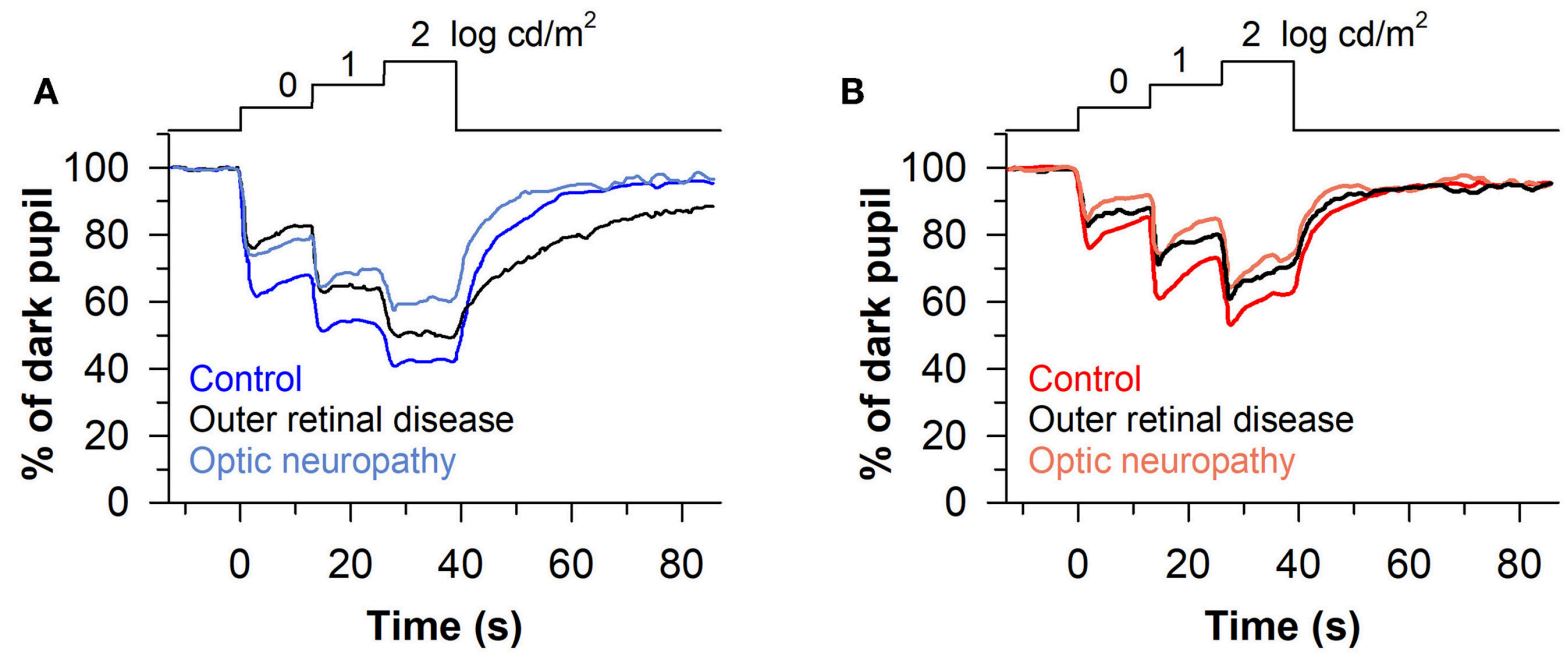
Protocol for assessing photoreceptor health using stepwise increases in light intensity. Pupillary responses were assessed in patients with outer retinal disease or optic nerve disease, using blue and red light stimuli that were presented for 13 s in each step (467 or 640 nm; 0, 1, and 2 log cd/m^2^). **(A)** Patients with outer retinal disease or optic nerve disease showed reduced pupillary light responses to each stepwise increase in blue light. However, the post-illumination pupillary light response after the last light step was prolonged only in patients with outer retinal disease. **(B)** Both groups of patients showed impaired pupillary responses to each stepwise increase in red light compared with controls, but there was no difference between groups in the PIPR. The figure is redrawn and modified with permission from (63).

The stepwise pupillometry protocol has also been compared between patients with degeneration of the outer retina (*n* = 23; retinitis pigmentosa, LCA, corneoretinal dystrophy of Bietti, cone-rod dystrophy, or Stargardt disease) and optic nerve disease (*n* = 13; ischemic optic neuropathy or compression lesion of the optic nerve) (63). Pupillary constriction responses to rod-, cone-, and melanopsin-weighted light stimuli were reduced in patients with either outer or inner retinal disease compared with healthy controls (Figures 8A,B). However, the pupillary re-dilation response after blue-light offset was much slower in patients with outer retinal disease compared with patients with optic nerve disease or healthy controls, which is consistent with other studies in which the stepwise light protocol was used (62, 68). In contrast, most studies that have used a 1-s flash of blue light to elicit the PIPR have found a relatively normal response after light offset in patients with outer retinal disease (60, 67, 72, 73), with only one study reporting a greater PIPR relative to controls (68). The difference in the PIPR across light exposure protocols may be related to the pre-exposure lighting conditions. In the stepwise light protocol, patients are exposed to continuously presented light and hence the PIPR is measured after light adaptation. Similar to results using the stepwise light protocol, a prolonged time course of pupillary re-dilation has been observed in patients with outer retinal disease after the offset of a bright blue light stimulus lasting 20 s or longer (i.e., after light adaptation) (38, 92). Even for a 1-s flash of bright blue light, the PIPR has been shown to be extended in patients with retinitis pigmentosa or LCA if the stimulus is presented after light adaption, rather than dark adaption (60). Together, these studies suggest that the stepwise pupillometry protocol can be used to differentiate loss of outer retinal function vs. melanopsin-dependent ipRGC function, by measuring transient pupillary responses at the start of each light step and the PIPR after light adaptation.

#### Assessing Photoreceptor Function Using Ramp-Up Light Protocols

**Figure 9:**
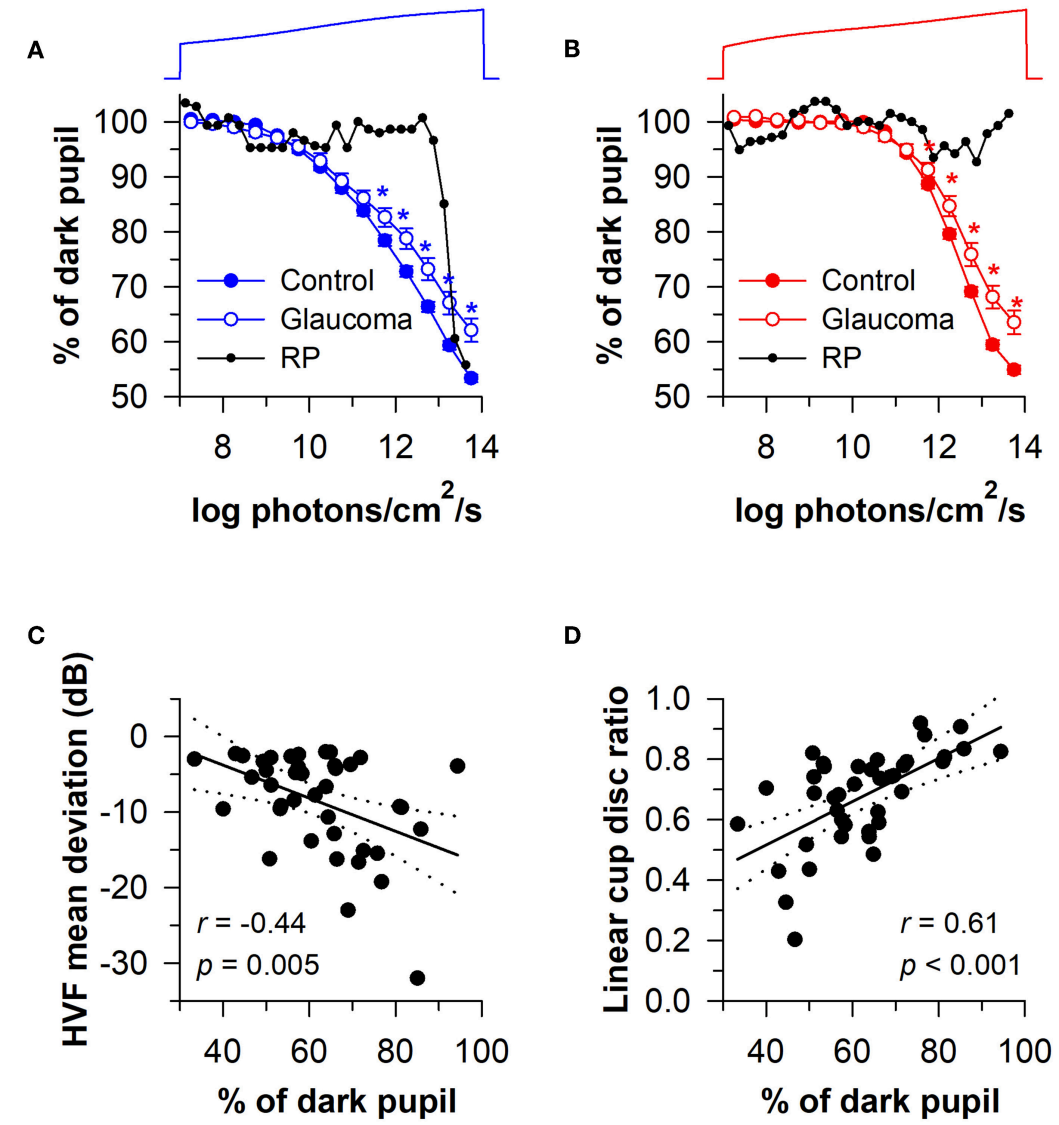
Protocol for assessing photoreceptor health using a ramp-up light exposure. Pupillary responses to blue light or red light were assessed during exposure to a continuously presented light stimulus that was increased gradually over a 2-min period (469 or 631 nm; from 7 to 14 log photons/cm^2^/s). **(A)** Pupillary responses to blue light were reduced in patients with glaucoma at higher irradiances compared with controls. In contrast, pupillary responses were reduced at dim-to-moderate light intensities in a patient with retinitis pigmentosa (RP) without rod/cone function, but were normal at the highest irradiances tested. **(B)** Pupillary responses to red light were also reduced in patients with glaucoma at higher irradiances, whereas there was no detectable response in the RP patient. In glaucomatous eyes, the magnitude of pupillary constriction during exposure to high-irradiance blue light (>13.5 log photons/cm^2^/s) correlated with **(C)** visual field loss determined by Humphrey Visual Field (HVF) mean deviation, and **(D)** optic disc cupping determined by Heidelberg Retinal Tomography. In **(C,D)**, the linear regression line is shown with 95% CIs. Data for glaucoma patients are replotted and modified with permission from (58). Data for the RP patient are replotted and modified with permission from (94).

Given that rods and cones are more sensitive to light than the melanopsin-dependent ipRGC response, photoreceptors in the outer retina and inner retina can be activated sequentially by using a gradually increasing light stimulus (i.e., a ramp-up light protocol). Based on irradiance-response curves for the pupillary light reflex, damage to the outer retina can be assessed by reduced pupillary responses to light stimuli at lower irradiances, i.e., below the threshold of activation for the melanopsin-dependent pupillary response (low-to-moderate intensity blue light or moderate-to-high intensity red light). By comparison, damage to the inner retina can be detected by measuring the pupillary response during exposure to continuous, high-intensity blue light. This approach has been tested using a modified Ganzfeld system to administer a gradually-increasing blue or red light stimulus (469 or 631 nm; corneal irradiance from 7 to 14 log photons/cm^2^/s) to one eye over a 2-min period (with the other eye covered), in order to construct irradiance-response curves for the direct pupillary light reflex (58). In patients with glaucoma (*n* = 40) with different stages of disease severity (Early, *n* = 19; Moderate, *n* = 10; Severe, *n* = 11), pupillary responses were impaired for the blue light stimulus at irradiances corresponding with the range of activation of the melanopsin-dependent response (>11.5 log photons/cm^2^/s), and the difference between patients and healthy controls was greatest at the highest irradiances tested (Figure 9A). Pupillary responses in glaucoma patients were also reduced for the red light stimulus at moderate-to-high intensities of red light (>11.5 log photons/cm^2^/s), suggesting reduced transmission from rod/cone photoreceptors to ipRGCs (Figure 9B). In contrast, the pupillary response in a patient with retinitis pigmentosa with no light perception was markedly reduced for the blue light stimulus at low-to-moderate light intensities (<13 log photons/cm^2^/s), and there was no detectable response to the red light stimulus (Figures 9A,B), which is consistent with loss of rod and cone function (94). However, the amplitude of his pupillary constriction response was in the normal range for high intensity blue light, suggesting preserved melanopsin-dependent ipRGC function. In the glaucoma patients, the magnitude of pupillary constriction to high-irradiance blue light (>13.5 log photons/cm^2^/s) was inversely correlated with Humphrey visual field mean deviation and optic disc cupping assessed using Heidelberg Retinal Tomography (Figures 9C,D) (58). These results suggest that ipRGC responses in glaucoma can be used to estimate damage to retinal ganglion cells that mediate image-forming vision.

In a later study, the ramp-up light exposure protocol was tested in a group of patients with early-stage glaucoma (*n* = 46; visual field mean deviation of −6 dB or better on automated perimetry) (95). Pupillary light responses were reduced in patients compared with healthy controls at moderate-to-high intensities of blue and red light (>11.0 log photons/cm^2^/s). In glaucomatous eyes, the maximum pupillary constriction amplitude correlated with RNFL thickness, but unsurprisingly, not with the amount of visual field loss. Hence, ipRGC dysfunction or cell loss can be detected in early stages of glaucoma and is associated with structural correlates of disease progression. In another study that used the ramp-up light protocol, patients with autosomal-dominant optic atrophy (*n* = 5) showed pupillary responses that were comparable to healthy controls (96), which is consistent with other studies that have found preserved ipRGC function in mitochondrial disease (77).

### Summary of Chromatic Pupillometry Methods Used to Assess Photoreceptor Health

Chromatic pupillometry methods can detect dysfunction of photoreceptors in diseases affecting either the outer retina or the inner retina. Clinical protocols that measure pupillary responses to 1-s light flashes allow for testing of rod function using a dim blue light stimulus after dark adaption, while cone function can be tested using a moderate-to-bright red light stimulus under light adaptation. This approach has been used to demonstrate loss of rod and/or cone function in outer retinal disease (e.g., retinitis pigmentosa and LCA). Melanopsin-dependent ipRGC function can be assessed by measuring the PIPR after exposure to a bright blue light stimulus. The PIPR after dark adaptation is reduced in patients with optic nerve disease (e.g., glaucoma and AION), with the notable exception of mitochondrial disease (e.g., LHON and autosomal dominant optic atrophy) in which ipRGCs appear to be preferentially spared. In stepwise light protocols, the phasic pupillary response to light stimulus onset, which is dominated by rods/cones, is impaired in outer retinal disease. In contrast, the sustained pupillary response to bright blue light, which is dominated by melanopsin, is impaired in diseases affecting ipRGC function, whereas the subsequent PIPR is prolonged in patients with outer retinal disease. In protocols in which light intensity is increased gradually over time (ramp-up light protocol), the tonic pupillary light reflex is impaired during exposure to dim-to-moderate intensity blue light or red light in outer retinal disease, whereas sustained pupillary constriction to high-irradiance blue light is impaired in inner retinal disease. In glaucoma, melanopsin-dependent pupillary responses correlate with visual field loss and anatomic correlates of optic nerve damage, suggesting that pupillometry methods can be used to estimate the degree of damage to ipRGCs and conventional retinal ganglion cells.

## Limitations and Considerations of Chromatic Pupillometry

Chromatic pupillometry is currently the only approach that can be used to assess rapidly the health of rods, cones, and melanopsin using a single light protocol. However, the contribution of outer retinal photoreceptors to phasic and tonic components of the pupillary light reflex is still not fully understood. Based on studies using the silent substitution method to selectively modulate the activity of different photoreceptor types, S-cones and M-cones may provide inhibitory input to ipRGCs that mediate the pupillary light reflex, whereas stimulation of L-cones and melanopsin induces pupillary constriction (55, 97, 98). A subset of melanopsin cells in the macaque retina has also been shown to exhibit an S-Off, (L+M)-On type of color-opponent receptive field (23). These findings have potential implications for interpreting the effects of blue light and red light stimuli on pupillary responses in chromatic pupillometry protocols, in which multiple photoreceptor types (i.e., rods, S-cones, M-cones, L-cones, and melanopsin) may be activated simultaneously and interact in complex ways at the level of ipRGCs. Outer retinal photoreceptors and melanopsin also differ in their time-course of dark adaptation and light adaptation (39, 99). Therefore, pre-exposure lighting conditions (e.g., the duration of prior darkness or exposure to light) can influence results of chromatic pupillometry testing (100, 101). Differences in the distribution of rods, cones, and melanopsin-containing retinal ganglion cells in the retina also gives rise to regional differences in spatial summation and ipRGC responses to light (102, 103). This may have implications for chromatic pupillometry protocols that administer light to different parts of the visual field to assess retinal health, e.g., central-field, hemi-field, quadrant-field, or annular light stimulation (84, 104–106).

Although chromatic pupillometry methods can be used to localize damage to the outer retina or the inner retina, they do not provide information on the specific disease type. For example, similar deficits in pupillary light responses are observed between patients with retinitis pigmentosa and LCA, and between patients with glaucoma or AION. Therefore, additional ophthalmic tests are required to establish an accurate diagnosis and its underlying pathophysiology. Pupillary light responses are also impaired in non-ocular diseases that result in demyelination or degeneration of the optic nerve, or altered autonomic nervous system function. For example, phasic pupillary light responses and the PIPR are reduced in patients with multiple sclerosis or Parkinson's disease (107–109). Efferent pathway defects from the midbrain to the pupils can also give rise to impaired pupillary constriction responses despite intact photoreceptor responses (110, 111). Moreover, the PIPR is reduced in patients with Seasonal Affective Disorder (SAD) or non-seasonal depression (112, 113), in whom there are no pathological changes in optic nerve function. These observations may be related to effects of the number of daylight hours on the amplitude of the PIPR (113, 114), perhaps through modulation of melanopsin protein levels and/or the sensitivity of ipRGCs to light. Relatedly, genetic variation in the melanopsin gene modulates pupillary responses to bright blue light and risk of SAD (112, 115–117). The PIPR and other pupillary light responses are also influenced by circadian phase (118, 119), sleep deprivation (119), and some types of medication (120). Additionally, cortical visual pathways are thought to be involved in modulating pupillary light responses (121, 122). Collectively, these studies show that results of chromatic pupillometry testing are influenced not only by the stimulation of retinal photoreceptors, but also by other biological pathways and disease processes.

An important concern regarding the use of chromatic pupillometry is whether it is necessary to adjust for effects of aging on pupillary responses. In aging, decreased sympathetic activity and smaller pupil size (i.e., age-related miosis) are thought to contribute to reduced pupillary constriction responses to light (123). When pupillary responses are measured relative to the dark-adapted pupil size, however, there is little effect of age on the amplitude of pupillary constriction (85, 124, 125). Similarly, age-dependent yellowing of the lens, which reduces transmission of short-wavelength light, does not appear to affect melanopsin-dependent pupillary responses. Rather, baseline-adjusted pupillary light responses are similar between young and older adults (124, 125), and the magnitude of the PIPR to blue light vs. red light is preserved across adulthood (85, 126, 127). Moreover, there is no effect of mild cataract (125) or narrow irido-corneal angles (128) on spectral responses of the pupil, and no effect of refractive error on the PIPR (127). Together, these results indicate that melanopsin-dependent pupillary responses are relatively stable in healthy aging. The pupillary light reflex also shows good test-retest reliability when assessed in the same individuals over short time intervals (68, 129). However, pupillary light responses in healthy individuals show substantial between-subject differences (85, 93), and usually overlap with responses in patients with mild-to-moderate retinal or optic nerve disease. Therefore, further optimization of chromatic pupillometry protocols may be necessary to differentiate reliably individuals with normal health vs. those with retinal or optic nerve disease.

A challenge in interpreting and comparing chromatic pupillometry studies is that different methods have been applied for measuring and delivering light to the eyes. In this review, we report light stimuli as they were described in the original research articles in which different units of light measurement were used (lux, cd/m^2^, and log photons/cm^2^/s). Units based on the photopic luminosity function (e.g., lux or cd/m^2^) are familiar to engineers and vision researchers, but should not be used for non-visual light responses including the pupillary light reflex (130). This is because it is well established that rods and melanopsin (not only cones) contribute to pupillary light responses in the photopic visual range, including sustained pupillary constriction and the PIPR. Instead, researchers should be encouraged to report either the power distribution (μW/cm^2^) or photon density (log photons/cm^2^/s) of their light stimulus, and/or calculate the α-opic illuminance values (α-opic lux) to provide an estimate of the effective illuminance for each of the 5 human photopigments. Doing so will make it easier to replicate experimental conditions across studies and to interpret the relative contribution of different photopigments to pupillary responses. Different types of light delivery systems have also been used in chromatic pupillometry studies. Most studies have administered light using a Ganzfeld system, or directed light through the pupil in Maxwellian view. While Ganzfeld systems are relatively easy to build and implement in pupillometry studies, the retinal illuminance is limited and cannot be specified by the experimenter for different stimulus conditions. In Maxwellian view, a high retinal illuminance field can be readily obtained and the size of the entry pupil can be controlled, but these systems require precise alignment of the eye, which is often achieved by using a bite bar to stabilize the participant's eye position (131). Hence, there are trade-offs that must be taken into consideration when choosing the type of light delivery system used for chromatic pupillometry.

Another challenge in comparing pupillometry studies is that different methods have been used for measuring pupillary responses. Some studies have measured the direct pupillary light reflex while covering the other eye (i.e., the pupil of the stimulated eye is recorded), whereas other studies have measured the consensual pupillary light reflex (i.e., the stimulated eye is dilated with a mydriatic agent and the pupil of the non-stimulated eye is recorded). Because the consensual light response is greater when the pupil of the stimulated eye is dilated, rather than constricted (132), the size of the pupil exposed to light must be taken into consideration when interpreting results of chromatic pupillometry. Additionally, many different types of pupillary response metrics have been used in chromatic pupillometry studies. For example, there are several metrics that have been used to quantify the PIPR, including the 6-s PIPR, area under the curve, re-dilation velocity, and the plateau of the PIPR based on the best-fit exponential model of the data. Some of these measures may be better than others at capturing the melanopsin-dependent component of the PIPR vs. the mixed contribution of rods/cones and melanopsin during the early phase of pupillary re-dilation. The reliability of these PIPR metrics has been shown to differ in healthy participants, with lower coefficients of variation for the 6-s PIPR and the plateau of the PIPR (50). Hence, some PIPR metrics are likely better than others, and this may also depend on the type of disease being examined. The PIPR amplitude also varies by stimulus duration, with shorter light stimuli (1 s) producing larger responses than longer light stimuli (10 or 30 s) (50). As such, a PIPR testing paradigm that utilizes a 1-s light stimulus and either a 6-s or plateau PIPR metric might prove most useful in clinical applications, as remains to be tested. Moving forward, researchers using chromatic pupillometry should strive to develop a set of consensus standards for light stimuli and pupillary response metrics that can be used to readily compare results across different studies and types of disease.

## Future Applications of Chromatic Pupillometry Methods

Early detection of retinal and optic nerve diseases is important for treating and preventing loss of vision. However, gradual loss of peripheral vision can go unnoticed for years. For example, patients with glaucoma often seek treatment after substantial and irreversible damage to the optic nerve has occurred. Based on results of chromatic pupillometry testing, patients with early-stage glaucoma show deficits in pupillary responses to bright blue light compared with healthy controls (84, 95). Such findings raise the possibility that pupillometry testing could be used to screen for early optic nerve dysfunction. An advantage of chromatic pupillometry methods is that they can be readily incorporated into portable testing systems for population screening. For example, chromatic pupillometry devices could be used in a polyclinic setting or in geriatric clinics to identify patients with suspected retinal or optic nerve disease. Such patients could then be directed to undergo a comprehensive ophthalmic examination to determine the origin of their impaired pupillary light response. Additionally, chromatic pupillometry methods can potentially be used in patients who have difficulty communicating or who are unable to follow procedures for visual field testing. Following diagnosis of the underlying condition, chromatic pupillometry testing could be used periodically to track progression of the disease and effects of treatment.

The pupillary light reflex can be used to test for intact melanopsin-dependent ipRGC responses in patients who are blind. Notably, a standard penlight examination is inadequate for this purpose, with results that are often unreliable (133). For example, pupillary light responses were studied extensively in a pair of blind patients with no light perception who were previously described as having no detectable pupillary light response based on penlight examination by an ophthalmologist (37, 38). Clinical testing of optic nerve function should therefore include conditions that are appropriate for assessing melanopsin-dependent responses, i.e., exposure to high-irradiance blue light on a background of darkness. In the future, this may be especially important for identifying blind patients with intact optic nerve function who should be considered as candidates for gene therapy trials to restore vision (see below). Additionally, melanopsin-dependent pupillary light responses could be used as a surrogate measure for other responses mediated by ipRGCs (119, 134, 135), in order to screen for blind patients with intact circadian photoreception who should expose themselves to light-dark cues to entrain to the 24-h solar day. Chromatic pupillometry methods can also be used to assess photoreceptor health in veterinary medicine, as demonstrated in dogs with sudden acquired retinal degeneration syndrome and optic nerve disease (136–138).

With the development of technologies for restoring vision in blind individuals, there is a need for standard clinical tests that can be used to help select suitable candidates and to estimate the degree of recovery of non-visual photoreception after treatment. Chromatic pupillometry methods may be useful for testing photoreceptor health in degenerative diseases (e.g., retinitis pigmentosa and LCA), in which mutations in photoreceptor-specific or non-photoreceptor-specific cells in the retina result in rod cell death, followed by loss of cones. Given that retinal ganglion cells and other retinal neuronal cell types can survive for long periods after blindness, vision can be partially restored by rendering the remaining cells photosensitive. This can potentially be achieved by surgically-implanted subretinal prostheses that can electrically stimulate retinal ganglion cells, injection of small-molecule photoswitches to bestow light sensitivity to retinal ganglion cells, and gene therapy to express light-regulated ion channels, transporters, or receptors (e.g., melanopsin or microbial opsins) in retinal neurons. These approaches have been tested in blind mice, demonstrating restoration of some behavioral light responses, and improved pupillary responses to light at low-to-moderate intensities (139–143). Similarly, gene therapy has been used to treat LCA in blind mice with impaired ability to regenerate visual photopigments (by restoring function of lecithin:retinol acyl transferase), which resulted in increased sensitivity of the pupillary light reflex by about 2.5 log units (144). Parallel findings have been reported for *RPE65*-associated LCA in humans, in whom viral delivery of the normal *RPE65* gene to the retina resulted in sustained improvement of subjective and objective measures of vision (145, 146), as well as an increase in sensitivity of pupillary responses to light that lasted for at least 3 years after follow-up. These studies demonstrate that gene therapy for restoring vision also results in improvement in the pupillary light reflex. In future studies, chromatic pupillometry protocols can potentially be used to quantify the degree of recovery of non-visual photoreceptor pathways in blind patients who undergo gene therapy or other treatments to restore vision.

In summary, chromatic pupillometry methods have the potential to improve detection and management of diseases affecting the retina or optic nerve. Previous studies have characterized the differential role of outer retinal photoreceptors and melanopsin in mediating the pupillary light reflex. This has led to development and testing of short-duration protocols for assessing pupillary responses in patients with retinal or optic nerve disease. Clinical studies have provided proof-of-concept that pupillometry can be used to localize loss of function to photoreceptors in the outer retina or inner retina in patients whose disease status was already known. We are now in the position to exploit these research findings to test prospectively the ability of chromatic pupillometry to detect abnormalities in ipRGC function. Future large-scale studies should therefore focus on optimizing, standardizing, and adapting chromatic pupillometry protocols for early detection of retinal and optic nerve diseases, and for monitoring disease progression or recovery after treatment.

## Data Availability Statement

This review article summarizes published data. Requests for datasets should be directed to the authors of the original research articles.

## Author Contributions

AR, DM, and JG made substantial contributions to the conception and/or design of the work, acquisition, analysis, and interpretation of data, drafting and/or revising the manuscript critically for important intellectual content. AR, DM, and JG provided final approval of the version to be published and agree to be accountable for all aspects of the work in ensuring that questions related to the accuracy or integrity of any part of the work are appropriately investigated and resolved.

### Conflict of Interest Statement

DM and JG filed a patent application for some of the chromatic pupillometry test procedures described in this manuscript. The remaining author declares that the research was conducted in the absence of any commercial or financial relationships that could be construed as a potential conflict of interest.
